# Misleading Information on the Properties of Vitamin C

**DOI:** 10.1371/journal.pmed.0020307

**Published:** 2005-09-27

**Authors:** Steve Hickey, Hilary Roberts

**Affiliations:** **1**Manchester Metropolitan UniversityManchesterUnited Kingdom; **2**ManchesterUnited Kingdom

The Cochrane review by Douglas et al. [1], which is referenced in the Best Practice article by Douglas and Hemilä [2], covers 60 years of research into vitamin C and the common cold. However, the review omits pharmacokinetic data that invalidate the conclusion that vitamin C is ineffective. This conclusion is not derivable from the data presented.

The dual-phase pharmacokinetics of vitamin C are described by the dynamic flow model [3,4]. Low gram-level intakes of ascorbate, leading to blood plasma levels below 70 μM/l, have a half-life of 8–40 days. Higher gram-level intakes have a plasma half-life of 30 minutes [3]. A large oral dose raises blood plasma levels briefly: they reach a peak after two to three hours, before decaying back to baseline. Frequent repeated doses allow sustained high plasma levels of about 250 μM/l [4,5].

Douglas and Hemilä reviewed intakes that transiently raise plasma ascorbate levels above 70 μM/l. A single dose does not raise the median level [6,7]. Daily supplements would, thus, not increase disease resistance to any great degree [3,4]. Single or double doses daily will not increase background plasma levels, regardless of the magnitude of the dose [6,7]. Since plasma ascorbate is at background level for the majority of the day, effects will be minimal.

There is widespread confusion about nutritional and pharmacological levels of supplementation [3]. Linus Pauling, typically, described nutritional gram-level doses able to provide a degree of disease prevention [8]. By contrast, pharmacological doses used for treatment are, at minimum, an order of magnitude larger and involve frequent doses. The doses should be at intervals of three hours or less [3]. Treatment doses are described by Cathcart's paper on titration to bowel tolerance [9]. To treat the onset of a cold, the therapy is perhaps a minimum of 10 g of oral ascorbic acid, followed by at least 2 g each hour [3,4].

Douglas and Hemilä give a misleading impression by not making it clear that the doses they consider are not pharmacological. They claim that the results of one study, giving an 8-g dose at the start of symptoms, are tantalising and deserve further assessment. However, once this single dose has been excreted, the protective effects will be lost. During illness, ascorbate is depleted rapidly and higher oral intakes are tolerated—up to 200 g per day [9]. It would be surprising if this 8-g dose had a large effect.

Studies on ascorbate require appropriate doses. Douglas and Hemilä have only confirmed that 60 years of vitamin C research has largely been wasted because of confusion between nutritional and pharmacological intakes, and because of a misunderstanding of the pharmacokinetics. It is essential that high-dose studies take into account ascorbate's dual-phase pharmacokinetics. The dosing regime should allow sustained high plasma levels to be achieved. The claim that vitamin C cannot prevent or cure the common cold is both premature and unwarranted.
